# Cathepsin E Is a Marker of Gastric Differentiation and Signet-Ring Cell Carcinoma of Stomach: A Novel Suggestion on Gastric Tumorigenesis

**DOI:** 10.1371/journal.pone.0056766

**Published:** 2013-02-22

**Authors:** Maki Konno-Shimizu, Nobutake Yamamichi, Ken-ichi Inada, Natsuko Kageyama-Yahara, Kazuya Shiogama, Yu Takahashi, Itsuko Asada-Hirayama, Mitsue Yamamichi-Nishina, Chiemi Nakayama, Satoshi Ono, Shinya Kodashima, Mitsuhiro Fujishiro, Yutaka Tsutsumi, Masao Ichinose, Kazuhiko Koike

**Affiliations:** 1 Department of Gastroenterology, Graduate School of Medicine, The University of Tokyo, Tokyo, Japan; 2 1st Department of Pathology, Fujita Health University School of Medicine, Aichi, Japan; 3 Second Department of Internal Medicine, Wakayama Medical College, Wakayama, Japan; The Chinese University of Hong Kong, Hong Kong

## Abstract

Gastric cancer (GC) presents various histological features, though the mechanism underlying its diversity is seldom elucidated. It is mainly classified into well differentiated tubular adenocarcinoma (tub1), moderately differentiated tubular adenocarcinoma (tub2), poorly differentiated adenocarcinoma (por), signet-ring cell carcinoma (sig), mucinous adenocarcinoma (muc), and papillary adenocarcinoma (pap). By screening, we found *cathepsin E (CTSE)* expresses universally in sig-type, occasionally in por-type, and rarely in tub1/tub2-type GC cell lines. In surgically-resected specimens, CTSE was immunostained in 50/51 sig-type (98.0%), 3/10 tub1-type (30.0%), 7/18 tub2-type (38.9%), 15/26 por-type (57.7%), 4/10 pap-type (40.0%), and 0/3 muc-type (0.0%) GC. In endoscopically-resected specimens, 6/7 sig-type (85.7%), 7/52 tub1-type (13.7%), 5/12 tub2-type (41.7%), 2/7 pap-type (28.6%) GC and 0/6 adenoma (0.0%) expressed CTSE. For non-malignant tissues, CTSE is universally expressed in normal fundic, pyloric, and cardiac glands of stomach, but hardly in other digestive organs. In the precancerous intestinal metaplasia of stomach, CTSE is mostly observed in mixed gastric-and-intestinal type and deficient in solely-intestinal type. CTSE expression is positively correlated with gastric marker MUC5AC (*p*<0.0001) and negatively correlated with intestinal marker MUC2 (*p* = 0.0019). For sig-type GC, in both tumors and background mucosa, expression of MUC5AC and CTSE is high whereas that of MUC2 is low, indicating that sig-type GC reflects the features of background mucosa. For gastric adenoma and tub1/tub2-type GC, more undifferentiated tumors tend to show higher expression of CTSE with MUC5AC and lower expression of MUC2 in tumors, but they tend to present lower expression of CTSE, MUC5AC and MUC2 in background mucosa. These suggest that more malignant gastric adenocarcinoma with stronger gastric and weaker intestinal properties tend to arise from background mucosa with decreased both gastric and intestinal features. In conclusion, CTSE is a marker of both gastric differentiation and signet-ring cell carcinoma, which should shed light on the mechanism of gastric tumorigenesis.

## Introduction

Gastric cancer (GC) is the fourth most common cancer and the second cause of cancer-related death worldwide, despite the gradual decrease of its incidence and mortality [1], [2]. It is well known that gastric malignancy presents various histological features [3]. The most widely used classification of gastric cancer is Lauren classification which distinguishes intestinal (differentiated) type GC and diffuse (undifferentiated) type GC [4]. Lauren typing is plain and easy to understand, though its oversimplification often leads to incapability of reflecting precise features of gastric malignancy [3]. In the more detailed Japanese classification of gastric cancer [5], which is similar to the WHO classification [6], gastric adenocarcinoma is classified into six categories: well differentiated tubular adenocarcinoma (tub1), moderately differentiated tubular adenocarcinoma (tub2), poorly differentiated adenocarcinoma (por), signet-ring cell carcinoma (sig), mucinouse adenocarcinoma (muc), and papillary adenocarcinoma (pap). Broadly speaking, Lauren’s diffuse (undifferentiated) type includes por-, sig-, and muc-type GC, whereas Lauren’s intestinal (differentiated) type comprises tub1-, tub2-, and pap-type GC [3].

Among the various histological GC types, we focused on signet-ring cell carcinoma (SRCC) of stomach (sig-type GC). SRCC is a mucin-secreting adenocarcinoma, in which intracytoplasmic mucin compresses their nuclei to give the cells characteristic “signet-ring” appearance [7]. While SRCC has been reported to be originated from various tissues, stomach is the most common organ of origin [7], [8]. Despite the frequent prevalence of SRCC among gastric malignancies, clinicopathological features of sig-type GC are still controversial; some previous studies reported that signet-ring cell histology presents a better prognosis [9], [10], [11], whereas other studies reported that histology of SRCC is a sign of rather worse prognosis [8], [12], [13], [14]. Under such background, we tried to find new specific marker genes for sig-type GC. In most previous studies [15], [16], [17], sig-type GC had been analyzed together with por-type and muc-type, because these three types of GC are categorized into Lauren’s diffuse type. However, many reports suggested that sig-type GC is quite different from por- or muc-type GC, from the viewpoint of molecular features or malignant potentials [3], [13], [18]. Therefore, we analyzed the sig-type GC independently from the por- and muc-type GC.

Among the vast number of genes, we screened several ones such as *cadherin* family genes [19], [20], human *mucin* genes [21], *vimentin* [22], *cathepsin* family genes [23], [24], [25], etc., whose expressions have been reported to be different between Lauren’s intestinal and diffuse type GC. The main purpose of our study is finding a clue to unresolved carcinogenesis of sig-type GC through identifying its specific marker genes. We expected that our trial would lead to unknown upstream regulatory genes (such as specific transcription factors) determining the properties of sig-type GC. Some transcription factors relating to gastrointestinal properties have been clarified such as *cdx* family genes [26], [27], *gli* family genes [28], and *sox2* [29], but we believe not a few crucial genes for gastrointestinal differentiation and gastric oncogenesis still remain undiscovered. Another purpose of our study is to analyze the very early stage of gastric tumorigenesis based on the expression of identified new marker genes. Not only focusing on sig-type GC, we further challenged to evaluate all types of early GC cases by analyzing identified marker gene expression in both the tumor lesion and adjacent mucosa. We are convinced our work should be a key to approaching the controversial features of sig-type GC, and also should be a lead to elucidating the various histological properties of gastric malignancy.

## Materials and Methods

### Cell Culture

Twenty gastric, ten colorectal, and two non-gastrointestinal cancer cell lines were maintained in DMEM with 10% fetal calf serum (Gibco/Invitrogen, Carlsbad, CA) at 37°C [30], [31]. All the cell lines were purchased from the American Type Culture Collection, RIKEN Cell Bank, or Cell Resource Center for Biomedical Research Institute of Development, Aging and Cancer, Tohoku University. Names and histological types of used cancer cell lines were described minutely in Supporting Document S1. For DNA demethylation reagent and histone deacetylase (HDAC) inhibitor, 5-Aza-2′-deoxycytidine (5-Aza-dC, Sigma-Aldrich) at 2 µg/ml or trichostatin A (TSA, Sigma-Aldrich) at 100 ng/ml were added to the culture medium.

### RT-PCR

Total cellular RNAs were prepared using the ISOGEN RNA isolation reaent (Wako, Osaka, Japan) as described previously [30]. Semi-quantitative RT-PCR was performed via the Superscript One-Step reaction using the Platinum Taq (Gibco/Invitrogen). The primer pairs and RT-PCR procedures employed to detect the expression levels of the *E-cadherin (CDH-1)*, *LI-cadherin (CDH-17)*, *MUC5AC*, *MUC6*, *MUC2*, *CTSE (Cathepsin E)*, *CTSD (Cathepsin D)*, *CTSB (Cathepsin B)*, *CTSL (Cathepsin L)*, and *GAPDH* are shown in the Table S1.

### Western Blotting

Whole cell extracts (20 µg each) lysed and boiled with 1x Sample buffer [30] were separated by electrophoresis on 12.5% SDS polyacrylamide gels, transferred to nitrocellulose membrane (Hybond-N, Amersham, Freiburg, Germany), and immunostained with anti-human Cathepsin E Antibody (#AF1294, R&D Systems, Minneapolis, MN) or anti-human β-Actin(C4) antibody (#sc-47778, Santa Cruz Biotechnology, Santa Cruz, CA). For second antibodies, horseradish peroxidase(HRP)-conjugated anti-goat IgG(H+L) donkey antibody (#705-035-003, Jackson, Baltimore, PA) and horseradish peroxidase(HRP)-conjugated anti-mouse IgG (H+L) goat antibody (#A90-216P, BETHYL, Montgomery, TX) were respectively used. Specific bands were detected with Immunostar LD (Wako, Osaka, Japan) and LAS-4000 (Fuji Film, Tokyo, Japan).

### Immunohistochemistry

Deparaffinization and endogenous peroxidase inactivation of clinical tissues were performed as described previously [3]. For CTSE, the primary immunostaining with anti-human CTSE goat polyclonal antibody (#AF1294, R&D Systems) at a 1∶100 dilution was performed for 16 hr at room temperature. After washing in PBS (Phosphate-Buffered-Salts) three times, the secondary immunostaining with Histofine Simple Stain MAX-PO(G) (Nichirei, Tokyo, Japan) was performed for 30 min at room temperature. After washing in PBS three times, the reaction products were visualized in 20 mg/dl 3,3′-diaminobenzidine tetrahydrochloride solution containing a drop of 30% H_2_O_2_, followed by wash with PBS. Nuclear counterstaining was accomplished with Mayer’s hematoxilin. For MUC5AC and MUC2, hydrated heating in 1 mM EDTA buffer (pH 8.0) at 120°C was first performed in a pressure cooker (Delicio 6L; T-FAL, Rumily, France) for 10 min for antigen retrieval. The primary immunostaining with anti-MUC5AC antibody (NCL-MUC-5AC, Novocastra, Newcastle-upon-Tyne, UK) at a 1∶200 dilution or anti-MUC2 antibody (NCL-NUC-2, Novocastra) at a 1∶500 dilution was performed for 16 hr at room temperature. After washing in PBS three times, the secondary immunostaining with Histofine Simple Stain MAX-PO(G) (Nichirei) was performed for 30 min at room temperature. The following step was the same as CTSE immunological staining. All the immunostained sections were evaluated independently by two pathologists, along with HE-stained and PAS-stained sections from the same lesions.

### Tumor Samples

Among the gastric cancer tissues banked at the Fujita Health University School of Medicine, we selected 51 gastric signet-ring cell carcinoma (sig) samples surgically resected. Furthermore, 67 gastric cancer surgical specimens of other five types of gastric adenocarcinoma (tub1, tub2, por, pap, and muc) were randomly selected from the same bank. For the endoscopically resected samples, we selected 84 specimens (78 gastric cancer cases and 6 gastric adenoma cases) that were treated in the University of Tokyo Hospital from 2005 to 2011. For all the clinical samples, written informed consents were obtained from the corresponding patients according to the Declaration of Helsinki. This study was approved by the institutional ethical review board for human investigation at Fujita Health University, and was also approved by the ethic committees of the University of Tokyo.

### Retrovirus Vectors

To generate retroviral vectors expressing *Cdx2*, *Gli1*, *Gli3*, *CTSE*, and *Sox2*, respective cDNAs were inserted into pMXs-IRES-puro (Cell Biolabs Inc., San Diego, CA) as follows. For *Cdx2*, pMXs-Cdx2-IRES-puro was generated as reported previously [26]. *Gli1* cDNA was obtained from pCR-XL-TOPO-Gli1 (Open Biosystems, Huntsville, AL) via PCR amplification using primers 5′-agccagatctatgagcccatctctgggattc-3′ and 5′-agtagcggccgcccctactctttaggcactagagt-3′. After double digestion with Bgl II and Not I, the obtained fragment was inserted into the BamH I/Not I site of pMXs-IRES-puro (Cell Biolabs Inc., San Diego, CA) to generate pMXs-Gli1-IRES-puro. *Gli3* cDNA was obtained from pCR-XL-TOPO-Gli3 (Open Biosystems) via PCR amplification using primers 5′-aatgcggccgctgatttccgttggt-3′ and 5′-tgcggatcctacgtgggcatttttg-3′. After double digestion with BamH I and Not I, the obtained fragment was inserted into the BamH I/Not I sites of pMXs-IRES-puro to generate pMXs-Gli3-IRES-puro. *CTSE* cDNA was obtained from pCMV-SPORT6-CTSE (Thermo Fisher Scientific, Madison, WI) via PCR amplification using primers 5′-ccgagatctatgaaacgctccttcttttg-3′ and 5′-gggctcgagtaaaactgtcgaatgaaga-3′. After double digestion with Bgl II and Xho I, the obtained fragment was inserted into the BamH I/Xho I sites of pMXs-IRES-puro to generate pMXs-CTSE-IRES-puro. *Sox2* cDNA was obtained from MKN-7 genomic DNA via PCR amplification using primers 5′-atgtacaacatgatggagacggagct-3′ and 5′-tcacatgtgtgagaggggcagtgtg-3′. The amplified fragment was inserted into the EcoR V sites of pT7Blue-T vector (Novagen, Darmstadt, Germany) to generate pT7Blue-Sox2. After double digestion with BamH I and Sal I, the obtained fragment was inserted into the BamH I/Xho I sites of pMXs-IRES-puro to generate pMXs-Sox2-IRES-puro. By cotransfection of these plasmids with vesicular stomatitis virus envelope G protein (VSV-G) expression plasmid into PLAT-GP prepackaging cell line (Cell Biolabs Inc.) respectively, VSV-G-pseudotyped MuLV-based retrovirus vectors expressing *Cdx2*, *Gli1*, *Gli3*, *CTSE*, and *Sox2* were prepared.

## Results

### Expression of *cathepsin E (CTSE)* is Significantly Correlated with Originated Histological Type of Gastric Cancer Cell Lines

**Figure 1:**
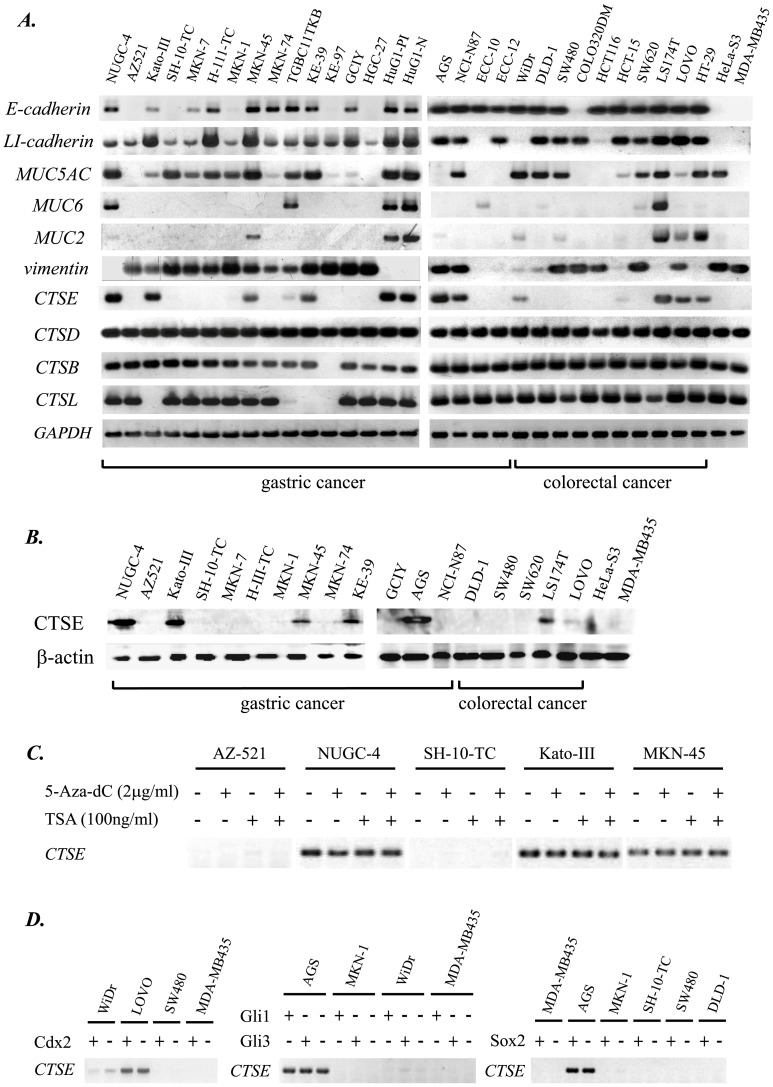
(A) Expression of *E-cadherin*, *LI-cadherin*, *MUC5AC*, *MUC6*, *MUC2*, *vimentin*, *CTSE*, *CTSD*, *CTSB, CTSL*, and *GAPDH* (internal control) mRNAs in a panel of 32 human cancer cell lines. 20 gastric, 10 colorectal, and 2 non-gastrointestinal cell lines (HeLa-S3 and MDA-MB435) were analyzed by RT-PCR. (B) Expression of CTSE protein in 13 gastric, 5 colorectal, and 2 non-gastrointestinal cancer cell lines analyzed by Western blotting. (C) RT-PCR detecting *CTSE* mRNA in 5 gastric cancer cells treated with 5-Aza-dC and/or TSA for 48 hours. (D) RT-PCR detecting *CTSE* mRNA in gastric (AGS, MKN-1, SH-10-TC), colorectal (WiDr, Lovo, SW480, DLD-1), and breast cancer (MDA-MB435) cell lines stably transduced with retroviral vector encoding *cdx2*, *gli1*, *gli3*, or *sox2* genes.

To search the marker genes for sig-type GC, we screened the expression of several genes reported to show distinctive expression between Lauren’s intestinal and diffuse type GC. Expression of *E-cadherin* (*CDH-1*) [19], *LI-cadherin* (*CDH-17*) [20], *MUC5AC* [21], *MUC6* [21], *MUC2* [21], *vimentin* [22], *CTSD* (*cathepsin D*) [23], [24], *CTSE* (*cathepsin E*) [23], [24], *CTSB* (*cathepsin B*) [25], *CTSL* (*cathepsin L*) [25], and *GAPDH* (internal control) genes were evaluated by RT-PCR in 20 GC lines together with the 12 cancer cell lines derived from other organs (Figure 1A). We then directed our attention to the *CTSE*, as it expresses in both Kato-III and NUGC-4 derived from sig-type GC, and also because it never expresses in MKN-7 and H-111-TC known to be originated from well differentiated tubular adenocarcinoma of stomach (tub1-type GC, Supporting Document S1).

For other examined genes, expression of *CDH-1* (*E-cadherin*), reported to be frequently deficient in Lauren’s diffuse type GC [19], [32], [33], was unexpectedly detected in the two sig-type GC-derived cells (Figure 1A). It was also unexpected that *CDH-17* (*LI-cadherin*), thought to be an intestinal marker gene [20], [26], [27], expresses in almost all the gastric cancer cell lines including sig-type (Figure 1A). For other cathepsin family genes, *CTSD* was reported to be highly expressed in diffuse type GC and also a prognostic parameter for gastric carcinoma patients [23], [34], but the results of RT-PCR revealed that all the examined cancer cell lines equally express *CTSD* (Figure 1A). *CTSB* and *CTSL* were reported to be elevated in gastric carcinoma [35] and correlated with its malignant property [36], but specific expression of neither *CTSB* or *CTSL* in sig-type GC-derived cell lines could be detected (Figure 1A). As a whole, the expression profile of *CTSE* seems to be quite different from other cathepsin family genes.

We next evaluated the relation between CTSE expression and histological type of 20 GC cell lines. Based on the detailed surveillance of literatures described in “Histological Type of Gastric Cancer Cell Lines” of Supporting Document S1, 19 GC cell lines could be sorted by Lauren and Japanese classification [3], [4], [5] (Table 1). According to the Lauren classification, 7 of 11 diffuse type GC-derived cells and 1 of 5 intestinal type GC-derived cells express CTSE (Table 1): *CTSE* gene tends to be expressed in diffuse type and deficient in intestinal type GC. Association between CTSE expression and histological feature of gastric malignancy gets clearer when more precise Japanese classification is applied. Among the cell lines from diffuse type GC, it is noteworthy that NUGC-4 and Kato-III originated from sig-type GC strongly express CTSE, whereas SH-10-TC and KE-97 originated from muc-type GC are deficient in CTSE expression. For cells derived from por-type GC, the most frequent histological type of gastric malignancy [3], CTSE expression is diverse: MKN-45, KE-39, HuG1-PI, HuG1-N, and AGS strongly express CTSE, whereas GCIY and HGC-27 are deficient in CTSE expression. On the contrary, among the cell lines from Lauren’s intestinal type GC, namely tub1 and tub2 of Japanese classification, MKN-7, H-111-TC, MKN-74, and AZ521 lack CTSE expression, with only one exception of NCI-N87 showing obvious CTSE expression.

**Table 1:** Summary of the association between CTSE (Cathepsin E) expression and original histological type of gastric cancer cell lines.

Gastric cancercell lines	Histological type based onJapanese classification	Histological type based onLauren classification	CTSE expression
SH-10-TC	muc	diffuse	–
KE-97	muc	diffuse	–
NUGC-4	sig	diffuse	+
Kato-III	sig	diffuse	+
MKN-45	por	diffuse	+
KE-39	por	diffuse	+
HuG1-PI	por	diffuse	+
HuG1-N	por	diffuse	+
AGS	por (tub2)	diffuse	+
GCIY	por	diffuse	–
HGC-27	por (muc)	diffuse	–
NCI-N87	tub1	intestinal	+
MKN-7	tub1	intestinal	–
H-111-TC	tub1	intestinal	–
MKN-74	tub1	intestinal	–
AZ-521	tub1 (tub2)	intestinal	–
(TGBC11TKB	N/A (tub2 ?)	intestinal ?	±)
MKN1	adenosquamous carcinoma		–
ECC-10	small-cell carcinoma (endocrine cell carcinoma)		–
ECC-12	small-cell carcinoma (endocrine cell carcinoma)		–

NOTE: Histological typing is based on the 3rd edition of Japanese Classification of Gastric Carcinoma. CTSE expression was evaluated due to the RT-PCR analysis.

From these result, we speculated that expression of CTSE is significantly correlated with histological type of gastric cancer. For other rare types of GC cell lines, MKN-1, ECC-10, and ECC-12 are absolutely deficient in CTSE expression (Figure 1A, Table 1).

### Expression of *cathepsin E (CTSE)* Gene is Regulated Majorly at the Transcription Level

Using the 13 gastric, 5 colorectal, and 2 other cancer cell lines, CTSE protein production was analyzed by Western blotting (Figure 1B). 7 of the 20 cell lines were also evaluated by immunohistochemistry (Figure S1). In the both analyses *CTSE* mRNA expression and CTSE protein production were mostly coupled, suggesting *CTSE* expression is mainly regulated at the transcriptional level. Besides, all-or-none expression of CTSE shown in RT-PCR, western blotting, and immunohistochemistry suggested that gastric cancer cells would be clearly classified into two categories: CTSE-expressing type and CTSE-deficient type.

To investigate the regulation of *CTSE* gene, two major epigenetic drugs, demethylating agent 5-Aza-2′-deoxycytidine and histone deacetylase inhibitor trichostatin A [37], were applied to five GC cell lines (Figure 1C). Three CTSE-expressing and two CTSE-deficient GC cell lines were treated, but we could not detect any change of *CTSE* transcription (Figure 1C). For methylation, we also searched CpG islands in the suggestive promoter region of human *CTSE* gene using two websites: “http://www.uscnorris.com/cpgislands2/cpg.aspx” demonstrating CpG island searcher and “http://www.ncbi.nlm.nih.gov” supported by the National Center for Biotechnology Information (NCBI). The results of both searches suggested that the promoter of human *CTSE* gene is characterized by a lower percentage of CpG dinucleotides (55%) and no CpG island, which are consistent with our results (Figure 1C).

In addition, we evaluated the effect of four transcription factors which have been reported to regulate many gastrointestinal genes: *cdx2* [26], [27], *gli* family genes (*gli1* and *gli3*) [28], and *sox2* [29]. Under the stable transduction of *cdx2*, *gli1*, *gli3*, and *sox2*, however, we could not detect an obvious change of *CTSE* expression (Figure 1D). At present, we could not elucidate the regulatory mechanism of *CTSE* gene, which should be resolved in the future.

### Cathepsin E (CTSE) is Definitely Expressed in Signet-ring Cell Carcinoma of Stomach

To validate the speculation from the GC cell lines (Table 1, Figure 1A), we next analyzed CTSE expression in clinical specimens. At first, 51 sig-type GC samples surgically resected were evaluated. Strikingly, obvious staining of CTSE was observed in all the 51 specimens examined, and in 50 of 51 cases, CTSE-positive cells occupied more than 90% of cancer cells in the tumor (Table 2).

**Table 2:** Summary of the association between CTSE (Cathepsin E) expression and histological type of gastric cancer using the surgical specimens.

Histological Typing of Gastric Cancer	4	3	2	1	Total
Signet-ring cell carcinoma (sig)	50	1	0	0	51
Well differentiated tubular adenocarcinoma (tub1)	1	2	3	4	10
Moderately differentiated tubular adenocarcinoma (tub2)	2	5	5	6	18
Poorly differentiated adenocarcinoma (por)	10	5	5	6	26
Papillary adenocarcinoma (pap)	0	4	3	3	10
Mucinous adenocarcinoma (muc)	0	0	3	0	3
Total	63	18	18	19	118

NOTE: Histological typing is based on the 3rd edition of Japanese Classification of Gastric Carcinoma. CTSE staining of cells in each gastric carcinoma was evaluated compared with stainig of CTSE-negative epithelial cells in the large intestine. Values assigned to the CTSE staining (from 1 to 4) were decided as follows: 1, percentage of cells with immunoreactivity of CTSE ranges from 0% to 10%; 2, percentage of cancer cells with immunoreactivity of CTSE ranges from 10% to 50%; 3, percentage of cancer cells with immunoreactivity of CTSE ranges from 50% to 90%; 4, percentage of cancer cells with immunoreactivity of CTSE is greater than 90%.

**Figure 2:**
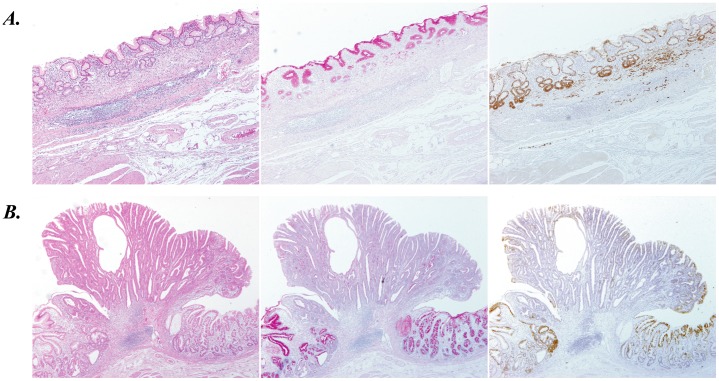
Immunostaining of CTSE in typical two types of gastric cancer; signet-ring cell carcinoma (A) and well-differentiated tubular adenocarcinoma (B). HE staining (left panel), PAS staining (middle panel), and immunostaining for CTSE (right panel) were shown in sequential sections of gastric cancer specimens, where no-cancerous adjacent gastric mucosa coexist.

Next, 67 surgical specimens derived from GC cases other than sig-type were analyzed, which presented various patterns of CTSE expression (Table 2). CTSE expression in por-type GC was obviously higher compared with other four types of GC, though it was much lower than that of sig-type GC (Table 2). Prevalence of CTSE positive cells in tub1-, tub2-, pap-, and muc-type GC is certainly lower, but it is not so low as other normal digestive organs (Table 2). Typical immunostaining images of CTSE in six types of GC are shown in Figure 2 (sig and tub1) and Figure S2 (tub2, pap, por, and muc).

Based on the result of surgical specimens, we further analyzed CTSE expression of 84 endoscopically resected gastric tumor samples. Compared with surgically specimens, the histology of endoscopically resected tissue is much more homogeneous; it was thence expected that association between CTSE expression and histology of gastric cancer could be analyzed more precisely. They comprised 78 cases of early stage adenocarcinoma (7 of sig-type, 52 of tub1-type, 12 of tub2-type, and 7 of pap-type GC) and 6 cases of precancerous adenoma (Table 3). CTSE expression of sig-, tub1-, tub2-, and pap-type GC in endoscopically resected tissues resembles to surgically resected ones with corresponding histology (Tables 2 and 3). Calculated CTSE expression scores (Table 3) clearly show a tendency that CTSE definitely expresses in sig-type GC whereas it tends to be deficient in tub1- and tub2-type GC. It should be also noted that gastric adenoma, which can be considered as more differentiated histological feature compared with tub1-type GC, showed the strongest deficiency of CTSE (Table 3).

**Table 3:** Summary of the association between CTSE (Cathepsin E) expression and histological type of gastric cancer using endoscopically resected specimens.

Histology of GastricCancer/Adenoma	Expression scores of CTSE				Total	Average of CTSE expression scores (1 to 4)
	4	3	2	1		
Sig	6	0	0	1	7	3.57±0.43
Adenoma	0	0	2	4	6	1.33±0.21
Tub1	3	4	17	28	52	1.65±0.12
Tub2	1	4	5	2	12	2.33±0.26
Pap	0	2	4	1	7	2.14±0.26
Total	10	10	28	36	84	1.93±0.11

NOTE: Histological typing is based on the 3rd edition of Japanese Classification of Gastric Carcinoma. CTSE staining of cells in each gastric tumor was evaluated compared with staining of CTSE-negative epithelial cells in the large intestine. Values assigned to the CTSE staining (from 1 to 4) were decided as follows: 1, percentage of cells with immunoreactivity of CTSE ranges from 0% to 10%; 2, percentage of cancer cells with immunoreactivity of CTSE ranges from 10% to 50%; 3, percentage of cancer cells with immunoreactivity of CTSE ranges from 50% to 90%; 4, percentage of cancer cells with immunoreactivity of CTSE is greater than 90%.

### CTSE is not only an Indicator of Signet-ring Cell Carcinoma of Stomach, but also a Gastric Differentiation Marker

To examine the distribution of CTSE in normal gut and precancerous stomach, immunostaining of CTSE was further applied to non-cancerous stomach and other digestive tract organs. In the stomach without atrophy and intestinal metaplasia, expression of CTSE is clearly observed in the fundic and pyloric glands of stomach (Figure S3D/S3A and S3E/S3B). Cardiac glands of stomach also express CTSE, though it is rather weaker than expression in fundic or pyloric glands (Figure S3F/S3C). Contrastively, CTSE is seldom expressed in esophagus, duodenum, small intestine, and large intestine (Figure S4).

**Figure 3:**
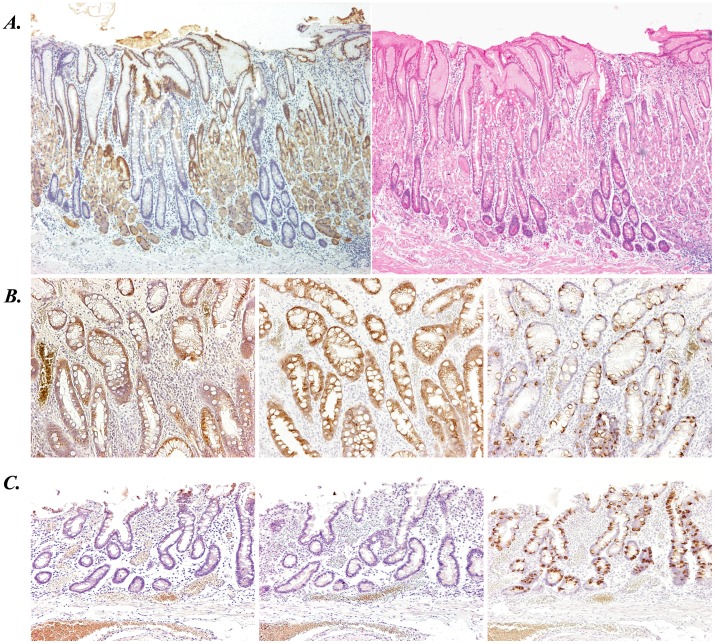
Expression of CTSE in non-malignant but precancerous gastric mucosa analyzed with immunohistochemistry. (A) CTSE immunostaining (left panel) and HE staining (right panel) of the stomach showing the mixture of normal fundic glands and intestinal metaplastic glands. (B, C) Immunostaining for CTSE (left), MUC5AC (middle), and MUC2 (right) in gastric intestinal metaplasia. Typical images of intestinal metaplasia with mixed gastric- and intestinal- feature (incomplete type, B) and solely intestinal feature (complete type, C) were shown.

We further evaluated CTSE expression in gastric mucosa with intestinal metaplasia, a well-known precancerous condition of stomach [26], [29], [38]. As shown in Figure 3A, gastric fundic glands and intestinal metaplastic glands exhibit contrastive staining of CTSE. Typically, intestinal metaplasia is classified into two categories: mixed gastric-and-intestinal type (incomplete type) and solely intestinal type (complete type) [29], [38]. It is well established that the former one expresses both MUC5AC (gastric marker mucin) and MUC2 (intestinal marker mucin), whereas the latter one expresses not MUC5AC but MUC2 [29]. In both types of intestinal metaplasia in stomach, we confirmed that expression of CTSE is similar to MUC5AC and opposite to MUC2 (Figure 3B and 3C).

To assess the association of CTSE expression with MUC5AC and MUC2 expression, their immunostaining was statistically evaluated using endoscopically resected 84 gastric tumor tissues (Table S2). The correlation analyses showed that CTSE expression is positively associated with gastric marker MUC5AC (*p*<0.0001) and negatively associated with intestinal marker MUC2 (*p* = 0.0019). Synthetically, we concluded that CTSE, like MUC5AC, is one of the gastric differentiation markers.

### More Undifferentiated Tubular Adenocarcinoma Tends to Arise from the Background Mucosa with Decreased Both “gastric” and “intestinal” Features

To investigate the initiation step of gastric tumorigenesis, the background mucosa of early cancer and adenoma was further evaluated, using 84 endoscopically resected specimens. CTSE expression of non-cancerous gastric mucosa adjacent to tumor lesion was evaluated, together with MUC5AC and MUC2 (Table 4). For sig-type GC, both the tumor lesion and background mucosa mostly showed strong expression of CTSE and MUC5AC, whereas expression of MUC2 was very weak in both of them (Table 4). Similar expression patterns of the three markers in the tumor and adjacent mucosa suggest that initiation of sig-type GC reflects the features of background mucosa, from the view of “gastric” and “intestinal” differentiation. That is to say, sig-type GC with non-intestinal gastric properties initially occurs from the background mucosa with non-intestinal and gastric features.

**Table 4:** Expression scores of CTSE, MUC5AC, and MUC2 (from 1 to 4 respectively) in gastric tumors cells (cancer or adenoma) and adjacent normal cells (non-tumorous epithelial cells of background mucosa).

Histology ofgastric tumors	CTSE intumor cells	CTSE in adjacentnormal cells	MUC5AC intumor cells	MUC5AC inadjacent normalcells	MUC2 intumor cells	MUC2 in adjacentnormal cells
Sig	3.57±0.43	3.86±0.14	3.57±0.43	3.29±0.18	1.43±0.43	1.43±0.30
Adenoma	1.33±0.21	3.17±0.31	1.67±0.42	3.67±0.21	1.83±0.31	3.17±0.31
Tub1	1.65±0.12	3.04±0.11	1.88±0.15	2.92±0.10	1.54±0.10	2.94±0.11
Tub2	2.33±0.26	2.17±0.11	2.08±0.31	2.75±0.25	1.42±0.19	2.58±0.29
Pap	2.14±0.26	2.57±0.20	3.14±0.40	2.86±0.14	1.86±0.26	3.29±0.42
Total	1.93±0.11	2.95±0.09	2.14±0.13	2.98±0.08	1.56±0.08	2.81±0.10

NOTE: Histology of gastric tumors was evaluated according to the 3rd edition of Japanese Classification of Gastric Carcinoma. Staining of CTSE, MUC5AC, and MUC2 in cells in each gastric tumor was evaluated compared with CTSE-deficient colorectal epithelium, MUC5AC-deficient colorectal epithelium, and MCU2-deficient normal gastric epithelium respectively. Values assigned to CTSE, MUC5AC, and MUC2 staining (from 1 to 4) were decided as follows: 1, percentage of cells with positive staining ranges from 0% to 10%; 2, percentage of cancer cells with positive staining ranges from 10% to 50%; 3, percentage of cancer cells with positive staining ranges from 50% to 90%; 4, percentage of cancer cells with positive staining is greater than 90%.

For gastric adenoma and tubular adenocarcinoma (tub1/tub2-type GC), contrastively, expression profiles of the three markers are very interesting (Table 4). More undifferentiated gastric tumors tend to increase expression of CTSE and MUC5AC in tumor lesions (tub2> tub1> adenoma) but decrease expression of these gastric markers in the background mucosa (tub2< tub1< adenoma). These suggest that more undifferentiated (hence more malignant) gastric tumors apt to show the stronger gastric property, whereas they tend to arise from the background mucosa with decreased gastric features. On the other hand, more differentiated gastric tumors tend to express MUC2 in both tumor lesions and background mucosa (adenoma>tub1> tub2). This suggests that intestinal differentiation of background gastric mucosa leads to the intestinally differentiated (hence less malignant) gastric tumors.

For pap-type GC, expressions of CTSE, MUC5AC, and MUC2 were considerably strong in both the tumor lesion and surrounding mucosa, which are quite different from the expression patterns of tub1/tub2-type GC (Table 4). Pap-type GC is classified into Lauren’s intestinal type together with tub1/tub2-type GC, but our present analyses suggested that pap-type and tub1/tub2-type GC should not treated in the same category, from the standpoint of gastric and intestinal features. In our previous reports analyzing *Brm* [3], a possible key marker gene of gut differentiation, expression of Brm in gastric papillary adenocarcinoma (pap) is quite different from tubular adenocarcinoma of stomach (tub1 and tub2). At present, we are convinced that histological difference between pap-type GC and tub1/tub2-type GC should be strictly recognized.

## Discussion

### Roles and Regulation of Cathepsin E (CTSE) in the Human Stomach

Cathepsin E (CTSE), a non-lysosomal intracellular aspartic protease, is one of the cathepsin family proteases [39], [40]. Another aspartic protease cathespin D (CTSD), a homologue of CTSE, represents a major proteolytic activity in the lysosomal component, but functional roles of CTSE have not been elucidated [24], [39]. Distribution of both proteinases is quite different: CTSD is universally existed in lysosomes of various tissues (consistent with the result in Figure 1A), whereas CTSE is mainly expressed in cells of the immune systems such as macropahges, lymphocytes, dendritic cells, etc [39]. Expression of CTSE in the stomach has also been reported [23], [24], though physiological and pathological function of gastric CTSE is currently unknown [39], [40]. In the present study evaluating as many as 202 clinical gastric samples, we clearly showed CTSE is both the gastric differentiation marker and the gastric signet-ring cell carcinoma marker, but the significance of gastric CTSE expression remains uncertain.

To analyze the relation of CTSE expression and oncogenic potential, we produced the MuLV-based retrovirus vector [26] carrying *CTSE* gene and transduced it into the CTSE-deficient gastric cancer cell lines: MKN-74, SH-10-TC, and MKN-1. We evaluated the possibility of altering gastric mucin production (Figure S5) or their morphological changes, but no alteration was observed. Using these established cell lines, we further performed both the colony formation in soft agar [30] and apoptosis induction by the treatment of actinomycin D, camptothecin, and staurosporine [41]. However, we could detect the effect of CTSE expression on neither anchorage independent growth nor resistance to drug-induced apoptosis (data not shown).

In the recent study, CTSE was reported to have some anti-oncogenic potential: Kawakubo *et al.* demonstrated that CTSE specifically induces growth arrest and apoptosis in human prostate cancer cell lines by catalyzing the proteolytic release of soluble tumor necrosis factor-related apoptosis-inducing ligand (TRAIL) from the cell surface [42]. However, CTSE-deficient mice did neither exhibit cancer-prone phenotype nor present obvious gastric disorders [43], [44], [45]. At present, it is a matter of conjecture whether reported antitumor activity of CTSE could apply gastric cancer including signet-ring cell carcinoma. Together with its unelucidated regulation and physiological function, effects of CTSE on gastric differentiation and tumorigenesis are fundamental problems which should be resolved in the future.

### Gastric Canceration from the Standpoint of “gastric” and “intestinal” Property of Background Mucosa

It has been widely accepted that “gastritis-atrophy-metaplasia-cancer” sequence is a main route for tubular adenocarcinoma of stomach [26], [29], [46]. Chronic infection of *Helicobacter pylori* is thought to be the major risk factor for such gastric tumorigenesis [1], [2]. Therefore, many studies have been executed to validate whether eradication of *Helicobacter pylori* could reduce the risk of gastric cancer [47], [48]. Most of these studies estimated the incidence of gastric cancer alone, but we think the histological types of gastric malignancies should be simultaneously evaluated. Preventing more undifferentiated (hence more malignant) cancer must be an essential standpoint for planning a strategy against gastric cancer.

Our result is suggestive for how to avoid the development of more undifferentiated gastric cancer. As shown in Table 4, more undifferentiated tubular adenocarcinoma tends to arise from the background mucosa with decreased gastric property. We therefore speculate that keeping the background mucosa from reducing “gastric” property would lead to prevention of more malignant cancer. Mainly due to chronic *Helicobacter pylori* infection, gastric property wanes along with progression of atrophic gastritis and intestinal metaplasia [1], [46]. It is thence anticipated that *Helicobacter pylori* eradication might lower the malignant potential of gastric cancer by maintaining the gastric property of background mucosa.

From the view of “intestinal” property, on the other hand, our result also suggest that more intestinal differentiation of background mucosa may lead to more differentiated (hence less malignant) tubular adenocarcinoma (Table 4). In Filipe’s classical study analyzing 1,525 subjects, it was reported that complete intestinal metaplasia (solely intestinal type) is associated with a low risk of gastric carcinogenesis, whereas incomplete type (gastric-and-intestinal type) denotes a tendency to stomach cancer [38]. Putting our result together, it is suggested that adequate intestinal differentiation of background mucosa can reduce the risk of tubular adenocarcinoma. That is, from the opposite point of view, insufficient intestinal differentiation (intestinal metaplasia) of gastric mucosa may lead to the more undifferentiated gastric tumors. *Helicobacter pylori* eradication would probably suppress the progression of intestinal differentiation of background mucosa, which might work negatively against prevention of the occurrence of more malignant (undifferentiated) gastric cancer.

It is clinically evident that gastric adenoma is much better than tub1-type GC, tub1-type GC is much better than tub2-type GC, and tub2-type GC is much better than por-type GC [49]. Therefore, we are convinced that clinical trial to lower malignant potential of gastric tumor is very important. For that purpose, detailed classification of gastric cancer is essential [5], [6], along with accurate estimation of background mucosa based on the balance of “gastric” and “intestinal” properties. We also believed that the effect of *Helicobacter pylori* eradication therapy on gasric malignancy should be reevaluated, from the standpoint of not only the tumor incidence but also the effect upon differentiation status of gastric cancer.

### Clinical Usefulness of CTSE Immunostaining in the Future

The meaning and regulatory mechanism of histology-specific CTSE expression in gastric cancer are still unknown. As was above-mentioned, alteration of oncogenic/anti-oncogenic potential in the CTSE-transduced GC cell lines could not be observed. We further analyzed expression of CTSE and depths of tumors in the 78 GC cases endoscopically resected (Table S3), but an obvious correlation could not be detected between them.

Nevertheless, strong CTSE expression in almost all sig-type GC cases and more than half of por-type GC cases should be clinically important (Table 2 and 3). These two histological types of GC, categorized into Lauren’s diffuse type, tend to infiltrate into the deeper layer of gastric wall without mass formation [4]. Therefore, scattering infiltration of sig- and por-type GC cells is often difficult to evaluate precisely. Actually, in the case shown in Figure 2A, a small amount of sig-type GC cells infiltrated in the submucosal layer were easily detected with CTSE immunostaining, but were hardly detected with HE staining or PAS staining. We expect that immunostaining of CTSE will be useful for detecting the scattered GC cells. Based on the present study, we are planning a clinical trial evaluating an efficiency of CTSE immunostaining for assessing the distribution of gastric cancer.

## Supporting Information

Figure S1 **Immunostaining of CTSE in seven cell lines originated from stomach or breast cancer.** Images of three CTSE-expressing gastric cancer cells (A: NUGC-4, B: Kato-III, C: AGS), three CTSE-deficient gastric cancer cells (D: SH-10-TC, E: GCIY, F: MKN-1), and CTSE-deficient breast cancer cell (G: MDA-MB435) were shown.
(TIF)

Figure S2 **CTSE immunostaining of four types of gastric adenocarcinoma.** HE staining (left panels) and CTSE immunostaining (right panels) are shown in sequential sections. (A, B) Moderately differentiated tubular adenocarcinoma (tub2). (C, D) Papillary adenocarcinoma (pap). (E, F) Poorly differentiated adenocarcinoma (por). (G, H) Mucinous adenocarcinoma (muc).
(TIF)

Figure S3 **CTSE immunostaining of three types of glands in the normal stomach.** HE staining (upper panels) and CTSE immunostaining (lower panels) are shown in sequential sections. (A, D) Fundic glands. (B, E) Pyloric glands. (C, F) Cardiac glands.
(TIF)

Figure S4 **CTSE immunostaining of other digestive organs than stomach.** Immunostaining of CTSE in normal esophagus (A), duodenum (B), small intestine (C), and colon (D) was demonstrated.
(TIF)

Figure S5 **RT-PCR detecting** ***MUC5AC*** **,** ***MUC6*** **,** ***CTSE*** **, and** ***GAPDH*** **mRNA in the** ***CTSE*** **-transduced MKN-74, SH-10-TC, and MKN-1 cells, all of which are originally deficient in CTSE expression.** These three gastric cell lines were infected with VSV-G pseudotyped MuLV-based retrovirus vectors expressing *CTSE* (+CTSE) or mock (+IP) to establish stable cell lines. NUGC4 was used as positive control for the above-mentioned four genes.
(TIF)

Table S1 **Primer pairs, annealing temperatures (Tm), and product sizes (Length) for the 11 genes analyzed by RT-PCR.**
(DOC)

Table S2 **A list of histological typing of analyzed 84 gastric cancer specimen endoscopically resected.** Values of CTSE, MUC5AC, and MUC2 expression in gastric cancer/adenoma and adjacent non-tumorous gastric mucosa are shown.
(DOC)

Table S3 **Association between the depth of tumors and CTSE expression in the 78 gastric cancer specimens endoscopically resected.** Depths of gastric cancers were classified into M (lesion confined to mucosal layer) or SM (lesion invading into the submucosal layer).
(DOC)

Supporting Document S1 **This file includes the supplementary materials and 18 supplementary references.**
(DOC)
