# Supplementary figures and images for: Cathepsin E Is a Marker of Gastric Differentiation and Signet-Ring Cell Carcinoma of Stomach: A Novel Suggestion on Gastric Tumorigenesis
Source: PLoS One. 2013 Feb 22;8(2):e56766. doi: 10.1371/journal.pone.0056766 (PMC3579941; doi:10.1371/journal.pone.0056766)

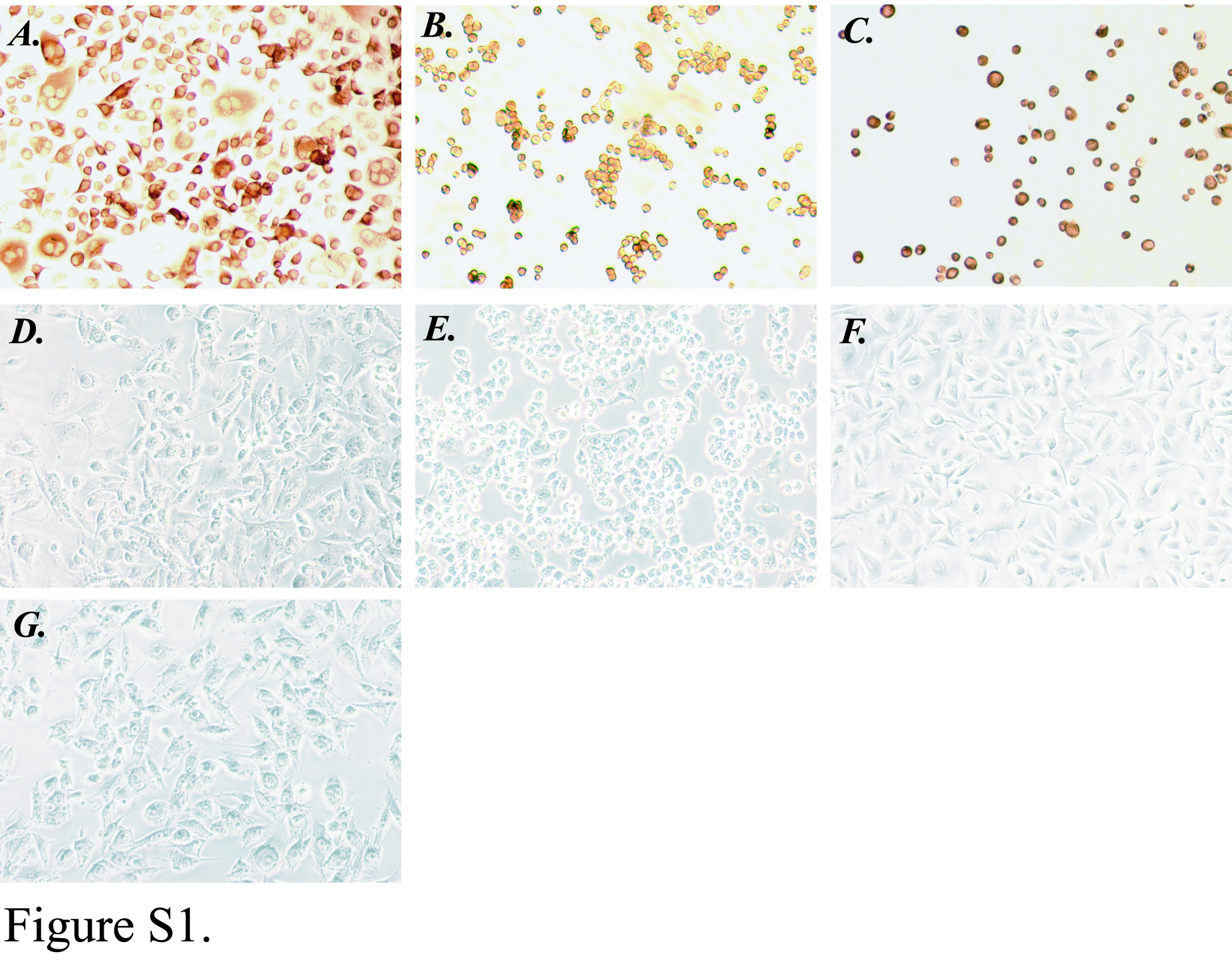

Supplement: Figure S1 — Immunostaining of CTSE in seven cell lines originated from stomach or breast cancer. Images of three CTSE-expressing gastric cancer cells (A: NUGC-4, B: Kato-III, C: AGS), three CTSE-deficient gastric cancer cells (D: SH-10-TC, E: GCIY, F: MKN-1), and CTSE-deficient breast cancer cell (G: MDA-MB435) were shown. (TIF) [file pone.0056766.s001.tif]

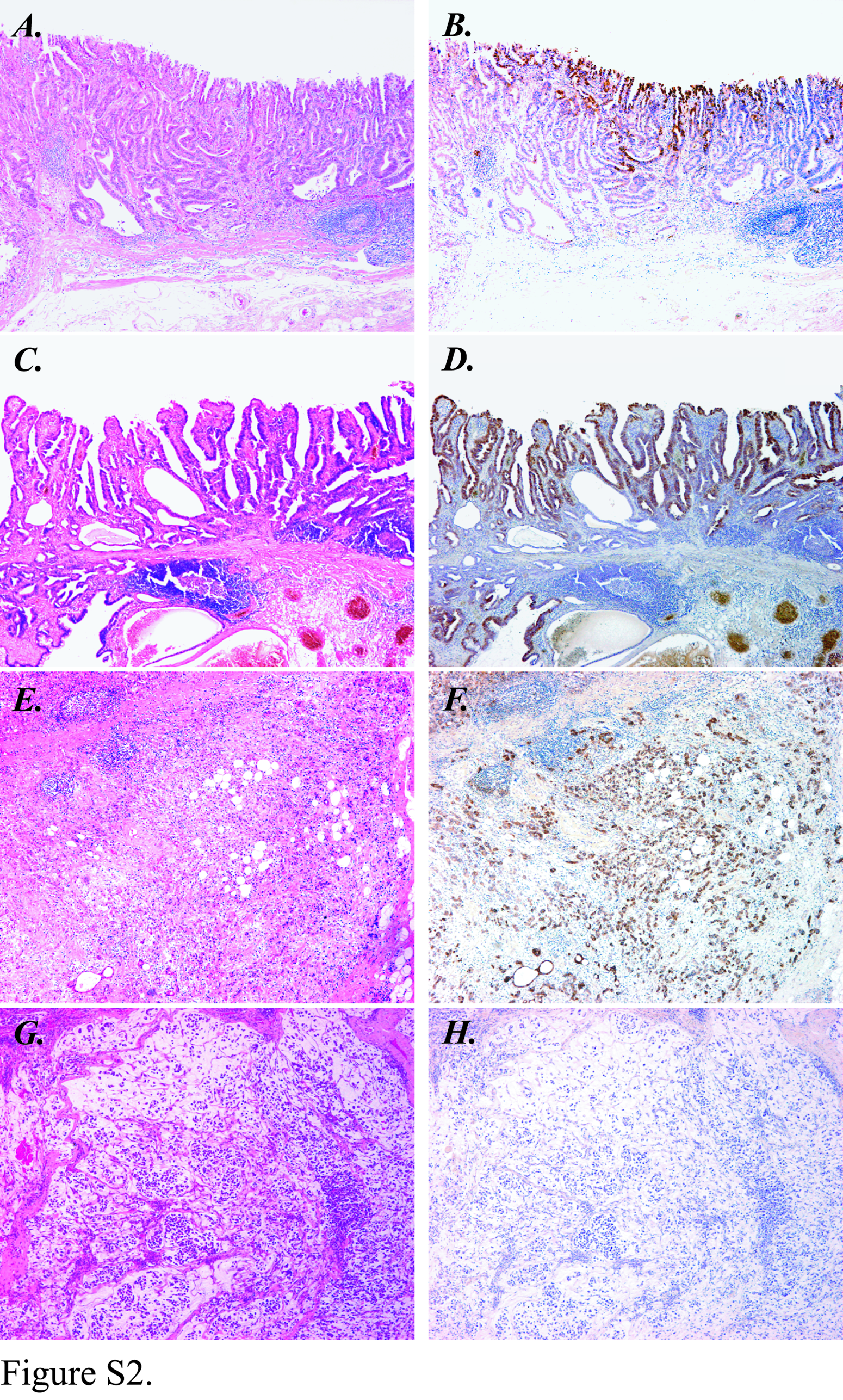

Supplement: Figure S2 — CTSE immunostaining of four types of gastric adenocarcinoma. HE staining (left panels) and CTSE immunostaining (right panels) are shown in sequential sections. (A, B) Moderately differentiated tubular adenocarcinoma (tub2). (C, D) Papillary adenocarcinoma (pap). (E, F) Poorly differentiated adenocarcinoma (por). (G, H) Mucinous adenocarcinoma (muc). (TIF) [file pone.0056766.s002.tif]

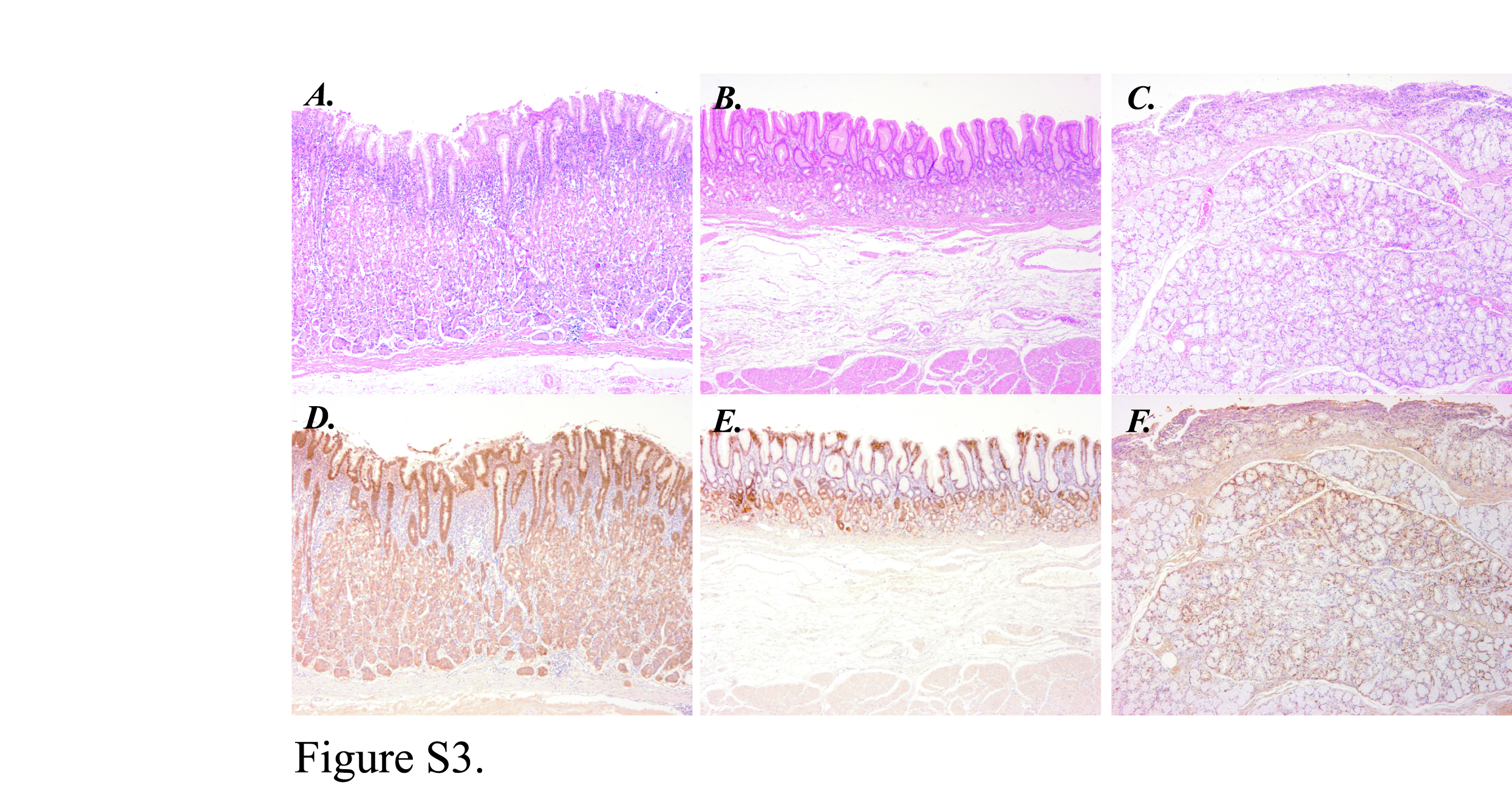

Supplement: Figure S3 — CTSE immunostaining of three types of glands in the normal stomach. HE staining (upper panels) and CTSE immunostaining (lower panels) are shown in sequential sections. (A, D) Fundic glands. (B, E) Pyloric glands. (C, F) Cardiac glands. (TIF) [file pone.0056766.s003.tif]

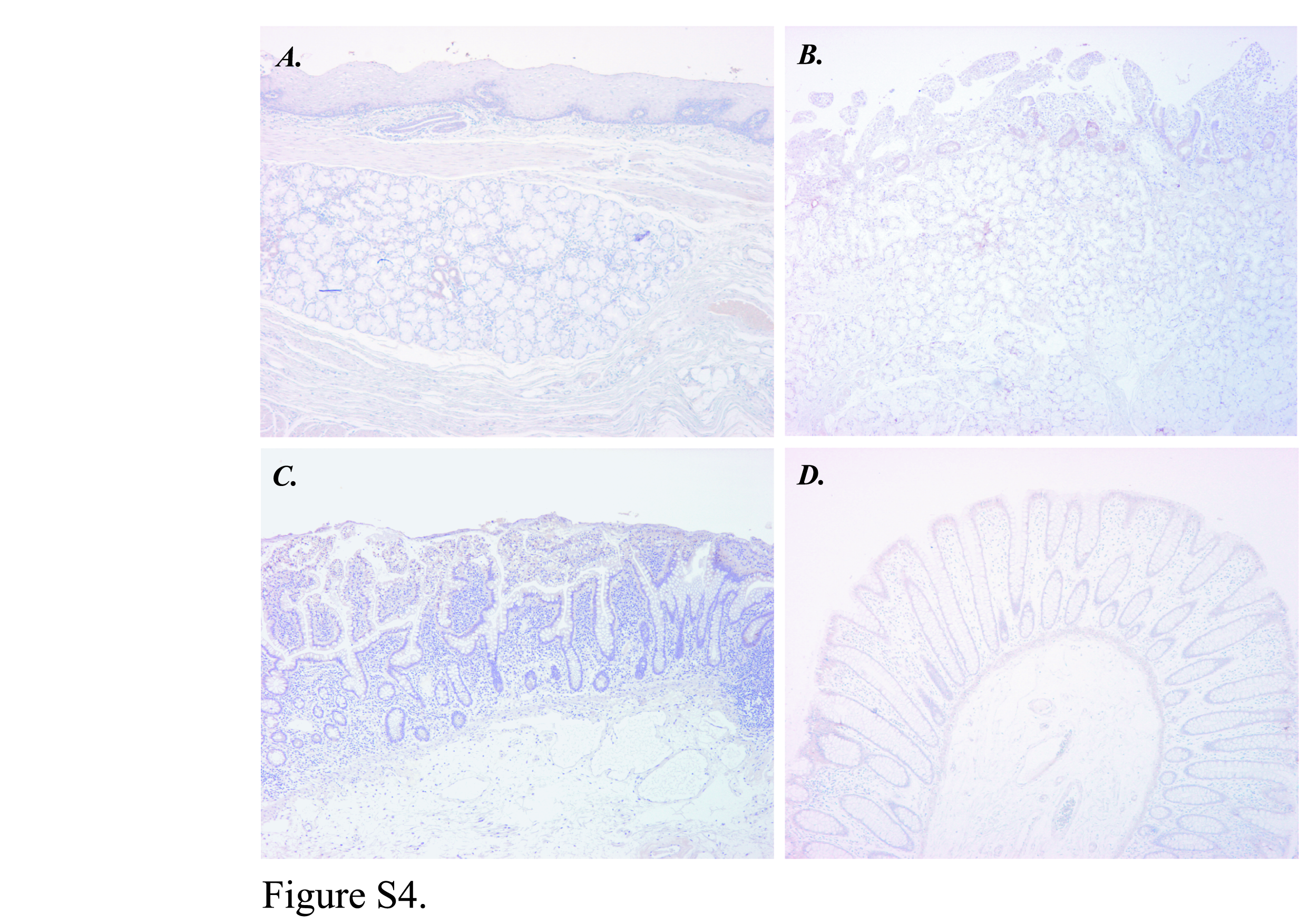

Supplement: Figure S4 — CTSE immunostaining of other digestive organs than stomach. Immunostaining of CTSE in normal esophagus (A), duodenum (B), small intestine (C), and colon (D) was demonstrated. (TIF) [file pone.0056766.s004.tif]

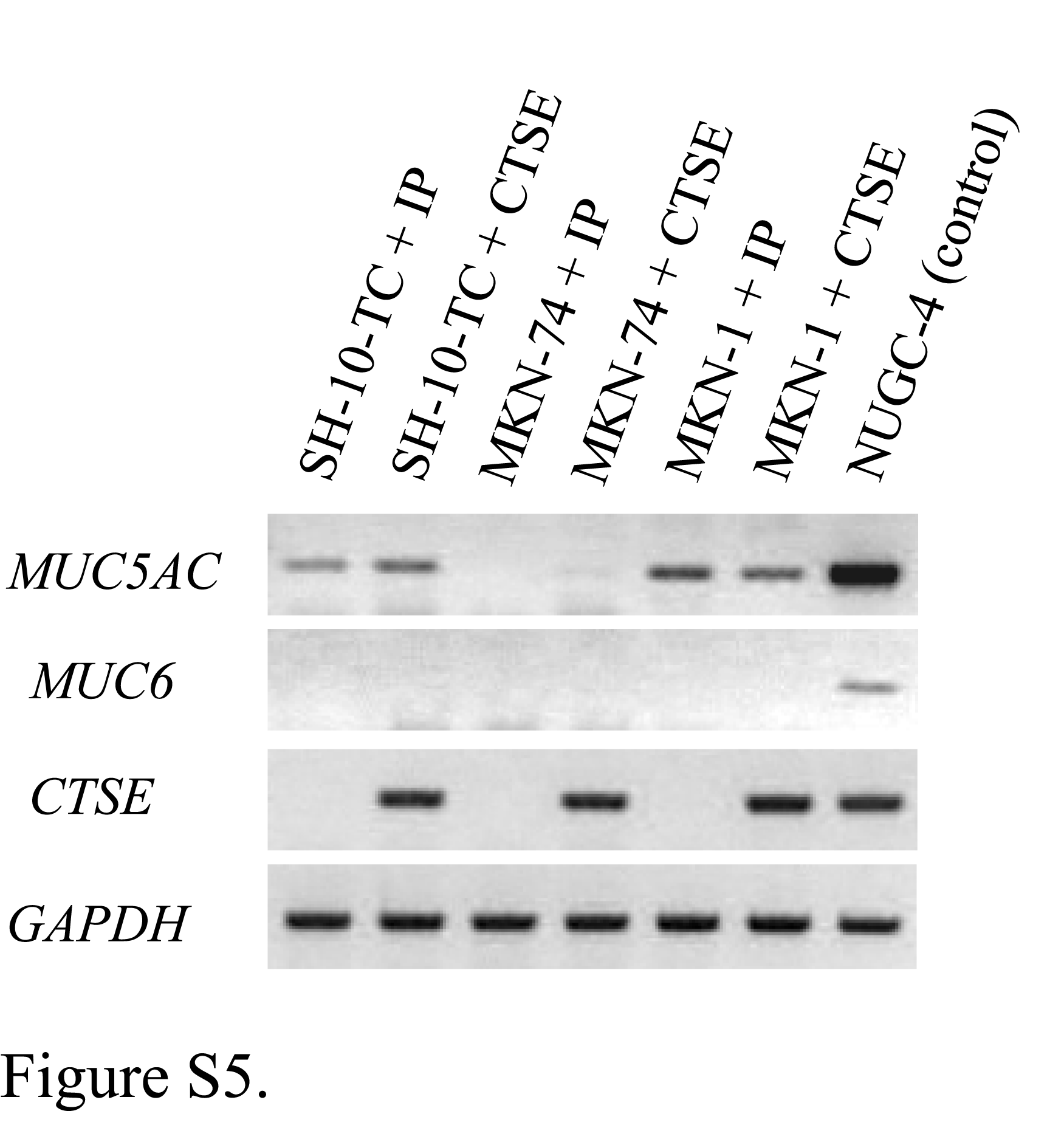

Supplement: Figure S5 — RT-PCR detecting MUC5AC , MUC6 , CTSE , and GAPDH mRNA in the CTSE -transduced MKN-74, SH-10-TC, and MKN-1 cells, all of which are originally deficient in CTSE expression. These three gastric cell lines were infected with VSV-G pseudotyped MuLV-based retrovirus vectors expressing CTSE (+CTSE) or mock (+IP) to establish stable cell lines. NUGC4 was used as positive control for the above-mentioned four genes. (TIF) [file pone.0056766.s005.tif]
