# Supplementary material for: Cathepsin E Is a Marker of Gastric Differentiation and Signet-Ring Cell Carcinoma of Stomach: A Novel Suggestion on Gastric Tumorigenesis
Source: PLoS One. 2013 Feb 22;8(2):e56766. doi: 10.1371/journal.pone.0056766 (PMC3579941; doi:10.1371/journal.pone.0056766)
Supplement: Table S1 — Primer pairs, annealing temperatures (Tm), and product sizes (Length) for the 11 genes analyzed by RT-PCR. (DOC) [file pone.0056766.s006.doc]

**Table S1.** Primer pairs, annealing temperatures (Tm), and product sizes (Length) for the 11 genes analyzed by RT-PCR.

| **Genes** | **Forward (F) and reverse (R) primer sequences** | **Tm(°C)** | **Length** |
| --- | --- | --- | --- |
| *E-cadherin (CDH-1)* | F: 5'-acctctgtgatggaggtcaca-3' | 58 | 544 |
|  | R: 5'-ccacattcgtcactgctacg-3' |  |  |
| *LI-cadherin (CDH-17)* | F: 5'-tgaaggccaagaaccgagtc-3' | 58 | 659 |
|  | R: 5'-ttttgatggggtgaggatcag-3' |  |  |
| *MUC5AC* | F: 5'-accggtgccacatgacggac-3' | 66 | 396 |
|  | R: 5'-acgtggccgcctcacacgtg-3' |  |  |
| *MUC6* | F: 5'-atggatggcagcagcaggcc-3' | 66 | 469 |
|  | R: 5'-tctggccgaagggtgtgatc-3' |  |  |
| *MUC2* | F: 5'-agccaccatggggctgccac-3' | 65 | 536 |
|  | R: 5'-tctgcaggccgttgtagtcc-3' |  |  |
| *vimentin* | F: 5'-gacaatgcgtctctggcacgtcttgaccttgaacgc-3' | 62 | 403 |
|  | R: 5'-gcatctggcgttccagggactcattggttc-3' |  |  |
| *CTSE* | F: 5'-tcctgctcaatggaccagagtg-3' | 58 | 418 |
|  | R: 5'-tcactcctcccacagccaagg-3' |  |  |
| *CTSD* | F: 5'-tccatccactgcaaactgctgg-3' | 58 | 469 |
|  | R: 5'-tcaggtaggacagagaacccttg-3' |  |  |
| *CTSB* | F: 5'-aaaggctctctcaacatcac-3' | 58 | 406 |
|  | R: 5'-ataagcttcccaaggtccag-3' |  |  |
| *CTSL* | F: 5'-aaaggctctctcaacatcac-3' | 58 | 442 |
|  | R: 5'-ataagcttcccaaggtccag-3' |  |  |
| *GAPDH* | F: 5'-accacagtccatgccatcac-3' | 58 | 423 |
|  | R: 5'-tccaccaccctgttgctgta-3' |  |  |
