# Supplementary material for: Cathepsin E Is a Marker of Gastric Differentiation and Signet-Ring Cell Carcinoma of Stomach: A Novel Suggestion on Gastric Tumorigenesis
Source: PLoS One. 2013 Feb 22;8(2):e56766. doi: 10.1371/journal.pone.0056766 (PMC3579941; doi:10.1371/journal.pone.0056766)
Supplement: Table S2 — A list of histological typing of analyzed 84 gastric cancer specimen endoscopically resected. Values of CTSE, MUC5AC, and MUC2 expression in gastric cancer/adenoma and adjacent non-tumorous gastric mucosa are shown. (DOC) [file pone.0056766.s007.doc]

**Table S2.** A list of histological typing of analyzed 84 gastric cancer specimen endoscopically resected. Values of CTSE, MUC5AC, and MUC2 expression in gastric cancer/adenoma and adjacent non-tumorous gastric mucosa are shown.

| **No.** | **Gastric Cancer typing** | **Value of CTSE expression in the tumor** | **Value of CTSE expression around the tumor** | **Value of MUC5AC expression in the tumor** | **Value of MUC5AC expression around the tumor** | **Value of MUC2 expression in the tumor** | **Value of MUC2 expression around the tumor** |
| --- | --- | --- | --- | --- | --- | --- | --- |
| **1** | **sig** | **4** | **4** | **4** | **3** | **1** | **2** |
| **2** | **sig** | **4** | **4** | **4** | **3** | **1** | **1** |
| **3** | **sig** | **4** | **3** | **4** | **3** | **1** | **3** |
| **4** | **sig** | **1** | **4** | **1** | **3** | **4** | **1** |
| **5** | **sig** | **4** | **4** | **4** | **3** | **1** | **1** |
| **6** | **sig** | **4** | **4** | **4** | **4** | **1** | **1** |
| **7** | **sig** | **4** | **4** | **4** | **4** | **1** | **1** |
| **8** | **tub1** | **2** | **2** | **2** | **3** | **2** | **4** |
| **9** | **tub1** | **1** | **3** | **1** | **3** | **2** | **2** |
| **10** | **tub1** | **2** | **2** | **4** | **3** | **2** | **2** |
| **11** | **tub1** | **1** | **4** | **1** | **3** | **2** | **3** |
| **12** | **tub1** | **2** | **3** | **1** | **4** | **2** | **2** |
| **13** | **tub1** | **2** | **3** | **3** | **3** | **2** | **3** |
| **14** | **tub1** | **1** | **3** | **1** | **3** | **3** | **3** |
| **15** | **tub1** | **1** | **4** | **1** | **3** | **4** | **2** |
| **16** | **tub1** | **3** | **3** | **4** | **3** | **2** | **3** |
| **17** | **tub1** | **1** | **2** | **1** | **2** | **1** | **3** |
| **18** | **tub1** | **2** | **4** | **2** | **3** | **1** | **2** |
| **19** | **tub1** | **2** | **4** | **2** | **3** | **1** | **2** |
| **20** | **tub1** | **3** | **3** | **3** | **3** | **2** | **1** |
| **21** | **tub1** | **1** | **3** | **2** | **3** | **1** | **4** |
| **22** | **tub1** | **1** | **3** | **1** | **2** | **1** | **3** |
| **23** | **tub1** | **2** | **1** | **1** | **2** | **1** | **4** |
| **24** | **tub1** | **1** | **4** | **1** | **3** | **1** | **4** |
| **25** | **tub1** | **1** | **3** | **1** | **4** | **1** | **4** |
| **26** | **tub1** | **1** | **2** | **1** | **3** | **3** | **3** |
| **27** | **tub1** | **1** | **3** | **1** | **3** | **2** | **2** |
| **28** | **tub1** | **2** | **3** | **1** | **3** | **2** | **2** |
| **29** | **tub1** | **1** | **2** | **2** | **3** | **2** | **3** |
| **30** | **tub1** | **4** | **3** | **3** | **2** | **1** | **2** |
| **31** | **tub1** | **1** | **3** | **1** | **3** | **1** | **3** |
| **32** | **tub1** | **1** | **3** | **1** | **3** | **1** | **3** |
| **33** | **tub1** | **4** | **4** | **2** | **3** | **1** | **2** |
| **34** | **tub1** | **1** | **4** | **2** | **4** | **1** | **3** |
| **35** | **tub1** | **2** | **4** | **3** | **3** | **1** | **4** |
| **36** | **tub1** | **2** | **4** | **4** | **4** | **1** | **3** |
| **37** | **tub1** | **1** | **4** | **1** | **3** | **2** | **3** |
| **38** | **tub1** | **3** | **4** | **4** | **3** | **1** | **3** |
| **39** | **tub1** | **1** | **3** | **2** | **3** | **2** | **3** |
| **40** | **tub1** | **4** | **3** | **2** | **2** | **1** | **4** |
| **41** | **tub1** | **2** | **2** | **1** | **3** | **2** | **4** |
| **42** | **tub1** | **1** | **3** | **3** | **3** | **2** | **3** |
| **43** | **tub1** | **2** | **3** | **1** | **3** | **1** | **4** |
| **44** | **tub1** | **1** | **1** | **1** | **2** | **2** | **3** |
| **45** | **tub1** | **1** | **3** | **1** | **2** | **1** | **3** |
| **46** | **tub1** | **1** | **4** | **3** | **4** | **1** | **3** |
| **47** | **tub1** | **2** | **3** | **3** | **4** | **2** | **4** |
| **48** | **tub1** | **3** | **3** | **2** | **2** | **1** | **3** |
| **49** | **tub1** | **2** | **3** | **1** | **2** | **1** | **4** |
| **50** | **tub1** | **1** | **3** | **1** | **3** | **2** | **4** |
| **51** | **tub1** | **1** | **4** | **1** | **4** | **1** | **1** |
| **52** | **tub1** | **1** | **1** | **1** | **1** | **1** | **4** |
| **53** | **tub1** | **2** | **4** | **1** | **4** | **1** | **3** |
| **54** | **tub1** | **2** | **3** | **4** | **3** | **1** | **2** |
| **55** | **tub1** | **1** | **3** | **2** | **4** | **1** | **2** |
| **56** | **tub1** | **1** | **3** | **1** | **3** | **3** | **3** |
| **57** | **tub1** | **1** | **2** | **1** | **1** | **2** | **3** |
| **58** | **tub1** | **1** | **3** | **4** | **3** | **1** | **3** |
| **59** | **tub1** | **2** | **4** | **4** | **3** | **1** | **3** |
| **60** | **tub2** | **2** | **2** | **3** | **2** | **1** | **3** |
| **61** | **tub2** | **4** | **2** | **1** | **2** | **1** | **3** |
| **62** | **tub2** | **1** | **2** | **4** | **4** | **2** | **1** |
| **63** | **tub2** | **2** | **2** | **2** | **2** | **1** | **3** |
| **64** | **tub2** | **2** | **2** | **1** | **2** | **1** | **3** |
| **65** | **tub2** | **2** | **2** | **3** | **4** | **3** | **1** |
| **66** | **tub2** | **3** | **2** | **1** | **2** | **1** | **2** |
| **67** | **tub2** | **3** | **2** | **1** | **3** | **1** | **3** |
| **68** | **tub2** | **3** | **3** | **2** | **3** | **2** | **4** |
| **69** | **tub2** | **2** | **2** | **3** | **3** | **2** | **2** |
| **70** | **tub2** | **3** | **3** | **3** | **4** | **1** | **2** |
| **71** | **tub2** | **1** | **2** | **1** | **2** | **1** | **4** |
| **72** | **pap** | **2** | **3** | **3** | **3** | **2** | **3** |
| **73** | **pap** | **2** | **3** | **3** | **3** | **1** | **3** |
| **74** | **pap** | **1** | **2** | **4** | **3** | **2** | **4** |
| **75** | **pap** | **2** | **2** | **4** | **3** | **3** | **4** |
| **76** | **pap** | **3** | **3** | **1** | **3** | **1** | **1** |
| **77** | **pap** | **3** | **2** | **3** | **2** | **2** | **4** |
| **78** | **pap** | **2** | **3** | **4** | **3** | **2** | **4** |
| **79** | **adenoma** | **1** | **3** | **1** | **4** | **3** | **3** |
| **80** | **adenoma** | **2** | **4** | **1** | **4** | **2** | **3** |
| **81** | **adenoma** | **2** | **4** | **3** | **3** | **1** | **4** |
| **82** | **adenoma** | **1** | **2** | **3** | **4** | **1** | **2** |
| **83** | **adenoma** | **1** | **3** | **1** | **4** | **2** | **3** |
| **84** | **adenoma** | **1** | **3** | **1** | **3** | **2** | **4** |
