# Supplementary material for: Cathepsin E Is a Marker of Gastric Differentiation and Signet-Ring Cell Carcinoma of Stomach: A Novel Suggestion on Gastric Tumorigenesis
Source: PLoS One. 2013 Feb 22;8(2):e56766. doi: 10.1371/journal.pone.0056766 (PMC3579941; doi:10.1371/journal.pone.0056766)
Supplement: Table S3 — Association between the depth of tumors and CTSE expression in the 78 gastric cancer specimens endoscopically resected. Depths of gastric cancers were classified into M (lesion confined to mucosal layer) or SM (lesion invading into the submucosal layer). (DOC) [file pone.0056766.s008.doc]

**Table S3.** Association between the depth of tumors and CTSE expression in the 78 gastric cancer specimens endoscopically resected. Depths of gastric cancers were classified into M (lesion confined to mucosal layer) or SM (lesion invading into the submucosal layer).

| **Depth of tumor invasion** | **Expression scores of CTSE** | | | | **Total** | **Average of CTSE**  **expression scores** |
| --- | --- | --- | --- | --- | --- | --- |
|  | **4** | **3** | **2** | **1** |  |  |
| M | 10 | 5 | 22 | 30 | 67 | 1.93 ± 0.13 |
| SM | 0 | 5 | 4 | 2 | 11 | 2.27 ± 0.69 |
