# Supplementary material for: Cathepsin E Is a Marker of Gastric Differentiation and Signet-Ring Cell Carcinoma of Stomach: A Novel Suggestion on Gastric Tumorigenesis
Source: PLoS One. 2013 Feb 22;8(2):e56766. doi: 10.1371/journal.pone.0056766 (PMC3579941; doi:10.1371/journal.pone.0056766)
Supplement: Supporting Document S1 — This file includes the supplementary materials and 18 supplementary references. (DOC) [file pone.0056766.s009.doc]

**Supplementary Materials**

**Cell Lines**

Twenty gastric cancer cell lines (NUGC-4, AZ521, KATO-III, SH-10-TC, MKN-7, H-111-TC, MKN-1, MKN-45, MKN-74, TGBC11TKB, KE-39, KE-97, GCIY, HGC-27, HuG1-PI, HuG1-N, AGS, NCI-N87, ECC-10, ECC-12), ten colorectal cancer cell lines (WiDr, DLD-1, SW480, COLO320DM, HCT116, HCT-15, SW620, LS174T, LOVO, HT-29), and two non-gastrointestinal cancer cell lines (HeLa-S3, MDA-MB435) were purchased from the American Type Culture Collection (ATCC), RIKEN Cell Bank (RCB), or Cell Resource Center for Biomedical Research (CRCBR) of Institute of Development, Aging and Cancer, Tohoku University. In detail, NUGC-4, AZ521, KATO-III, SH-10-TC, H-111-TC, MKN-7, GCIY, and Hct-15 were from CRCBR; MKN1, MKN45, MKN74, TGBC11TKB, KE-39, KE-97, HGC-27, HuG1-PI, HuG1-N, ECC-10, ECC-12 cell lines were from the RCB; and AGS, NCI-N87, WiDr, DLD-1, SW480, HCT116, SW620, LS174T, HT-29, COLO320DM, LOVO, HeLa-S3, and MDA-MB435 were from ATCC. All the cell lines were maintained in DMEM with 10% fetal calf serum (Gibco/Invitrogen, Carlsbad, CA) at 37°C as described previously(1, 2).

**Histological Type of Gastric Cancer Cell Lines**

NUGC-4(3) and Kato-III(4-6) were reported to be originated from signet-ring cell carcinoma of the stomach (sig-type). MKN-7(5, 7), H-111-TC(8, 9), MKN-74(5-7, 10), and NCI-N87(10, 11) were reported to be originated from well differentiated tubular adenocarcinoma of the stomach (tub1-type). AZ521, reported to be derived from tubular adenocarcinoma of the stomach(6), was therefore originated from well or moderately differentiated tubular adenocarcinoma of the stomach (tub1-type or tub2-type). SH-10-TC(9) and KE-97(12) were originated from mucinous adenocarcinoma of the stomach. MKN-45(4-6), KE-39(13), HuG1-PI(13), HuG1-N(13), GCIY(14, 15), HGC-27(15, 16), and AGS(17) were reported to be originated from poorly differentiated adenocarcinoma of the stomach (por-type). For other rare types of gastric cancer cell lines, MKN-1 derived from adenosquamous carcinoma(6, 10); ECC-10 and ECC-12(18) derived from gastric small-cell endocrine carcinoma. Among the used 20 gastric cell lines, we could not find a clear description identifying the histological type of TGBC11TKB in literatures, though it seemed to be originated from tub2-type GC.

**Primer pairs for RT-PCR**

The primer pairs and RT-PCR procedures employed to detect the expression levels of the eleven genes are shown in the Table S1.

**Supplementary References**

1. Yamamichi N, Yamamichi-Nishina M, Mizutani T*, et al.* The Brm gene suppressed at the post-transcriptional level in various human cell lines is inducible by transient HDAC inhibitor treatment, which exhibits antioncogenic potential. *Oncogene* 2005 Aug 18; 24(35): 5471-81.

2. Yamamichi N, Inada K, Ichinose M*, et al.* Frequent loss of Brm expression in gastric cancer correlates with histologic features and differentiation state. *Cancer Res* 2007 Nov 15; 67(22): 10727-35.

3. Kobayashi M, Iwamatsu A, Shinohara-Kanda A, Ihara S, Fukui Y. Activation of ErbB3-PI3-kinase pathway is correlated with malignant phenotypes of adenocarcinomas. *Oncogene* 2003 Mar 6; 22(9): 1294-301.

4. Oda T, Kanai Y, Oyama T*, et al.* E-cadherin gene mutations in human gastric carcinoma cell lines. *Proc Natl Acad Sci U S A* 1994 Mar 1; 91(5): 1858-62.

5. Motoyama T, Hojo H, Watanabe H. Comparison of seven cell lines derived from human gastric carcinomas. *Acta Pathol Jpn* 1986 Jan; 36(1): 65-83.

6. Yamaoka N, Yamaguchi T, Otsuji E*, et al.* In vitro reactivity and in vivo biodistribution of the monoclonal antibody A7 using human gastric carcinoma cell lines. *Br J Cancer* 1994 Sep; 70(3): 405-8.

7. Kameda T, Yasui W, Yoshida K*, et al.* Expression of ERBB2 in human gastric carcinomas: relationship between p185ERBB2 expression and the gene amplification. *Cancer Res* 1990 Dec 15; 50(24): 8002-9.

8. Yanagihara K, Ito A, Toge T, Numoto M. Antiproliferative effects of isoflavones on human cancer cell lines established from the gastrointestinal tract. *Cancer Res* 1993 Dec 1; 53(23): 5815-21.

9. Nosoh Y, Toge T, Niimi K*, et al.* Experimental studies on the combined effects of alpha and gamma interferons against human tumor xenografts transplanted into nude mice. *Jpn J Surg* 1987 May; 17(3): 168-73.

10. Mayer B, Klement G, Kaneko M*, et al.* Multicellular gastric cancer spheroids recapitulate growth pattern and differentiation phenotype of human gastric carcinomas. *Gastroenterology* 2001 Oct; 121(4): 839-52.

11. Park JG, Frucht H, LaRocca RV*, et al.* Characteristics of cell lines established from human gastric carcinoma. *Cancer Res* 1990 May 1; 50(9): 2773-80.

12. Uesugi H, Atari E. [Establishment and pathological study of a new poorly differentiated mucinous gastric cancer cell line]. *Nihon Shokakibyo Gakkai Zasshi* 1995 Jan; 92(1): 19-25.

13. Uesugi H, Yamashita K, Atari E*, et al.* [Establishment and characterization of strain KE-39 derived from human gastric cancer]. *Hum Cell* 1994 Dec; 7(4): 227-32.

14. Yokoyama H, Nakanishi H, Kodera Y*, et al.* Biological significance of isolated tumor cells and micrometastasis in lymph nodes evaluated using a green fluorescent protein-tagged human gastric cancer cell line. *Clin Cancer Res* 2006 Jan 15; 12(2): 361-8.

15. Tamura G, Sakata K, Nishizuka S*, et al.* Inactivation of the E-cadherin gene in primary gastric carcinomas and gastric carcinoma cell lines. *Jpn J Cancer Res* 1996 Nov; 87(11): 1153-9.

16. Akagi T, Kimoto T. Human cell line (HGC-27) derived from the metastatic lymph node of gastric cancer. *Acta Med Okayama* 1976 Jun; 30(3): 215-9.

17. Moon WS, Tarnawski AS, Chai J, Yang JT, Majumdar AP. Reduced expression of epidermal growth factor receptor related protein in gastric cancer. *Gut* 2005 Feb; 54(2): 201-6.

18. Fujiwara T, Motoyama T, Ishihara N*, et al.* Characterization of four new cell lines derived from small-cell gastrointestinal carcinoma. *Int J Cancer* 1993 Jul 30; 54(6): 965-71.
